# Supplementary figures and images for: Polygenic prediction of cardiorespiratory fitness in the Trøndelag health study (HUNT)
Source: Sci Rep. 2025 Dec 16;16:184. doi: 10.1038/s41598-025-28894-7 (PMC12764957; doi:10.1038/s41598-025-28894-7)

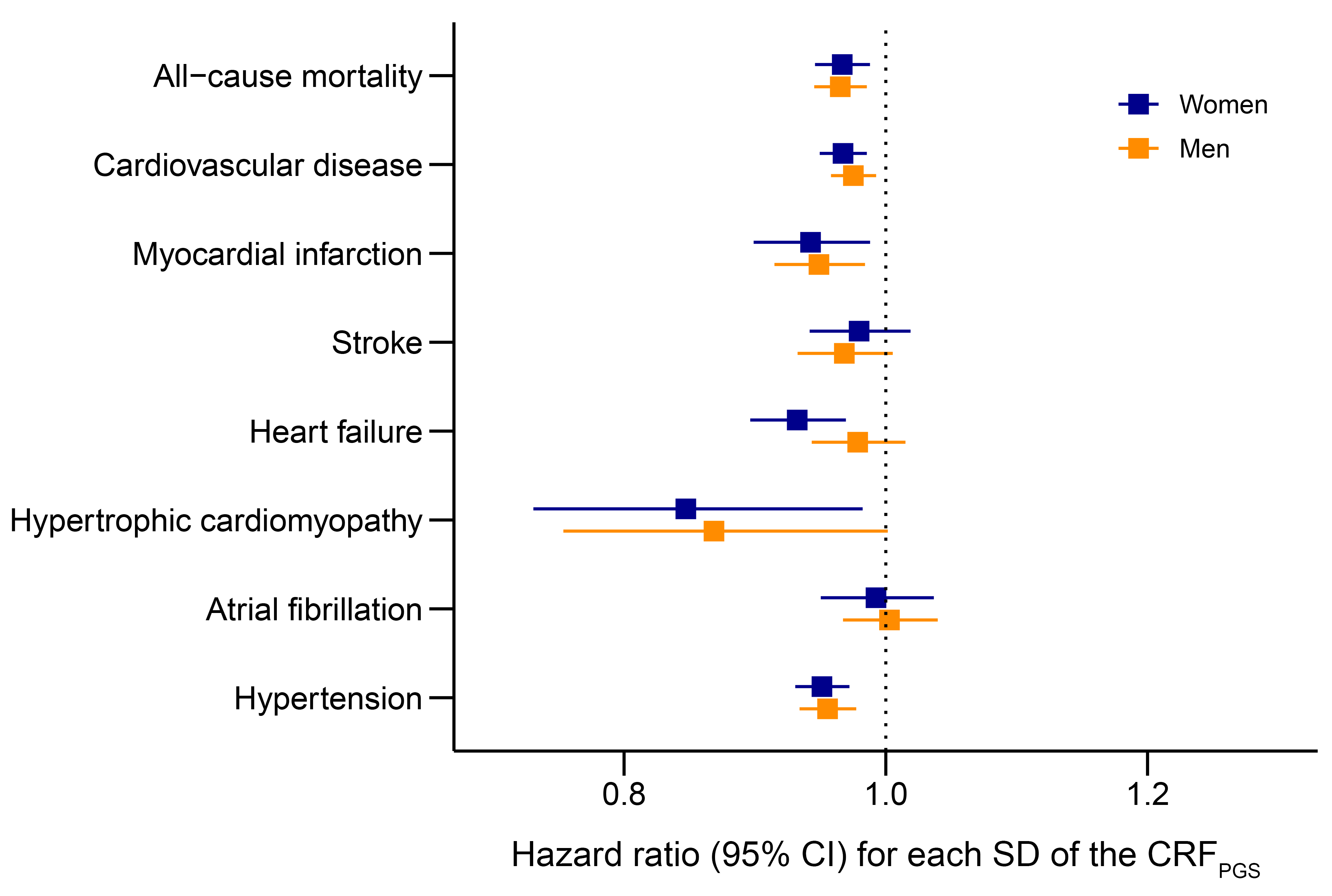

Supplement: Supplementary file 1 — Supplementary Material 1 [file 41598_2025_28894_MOESM1_ESM.png]

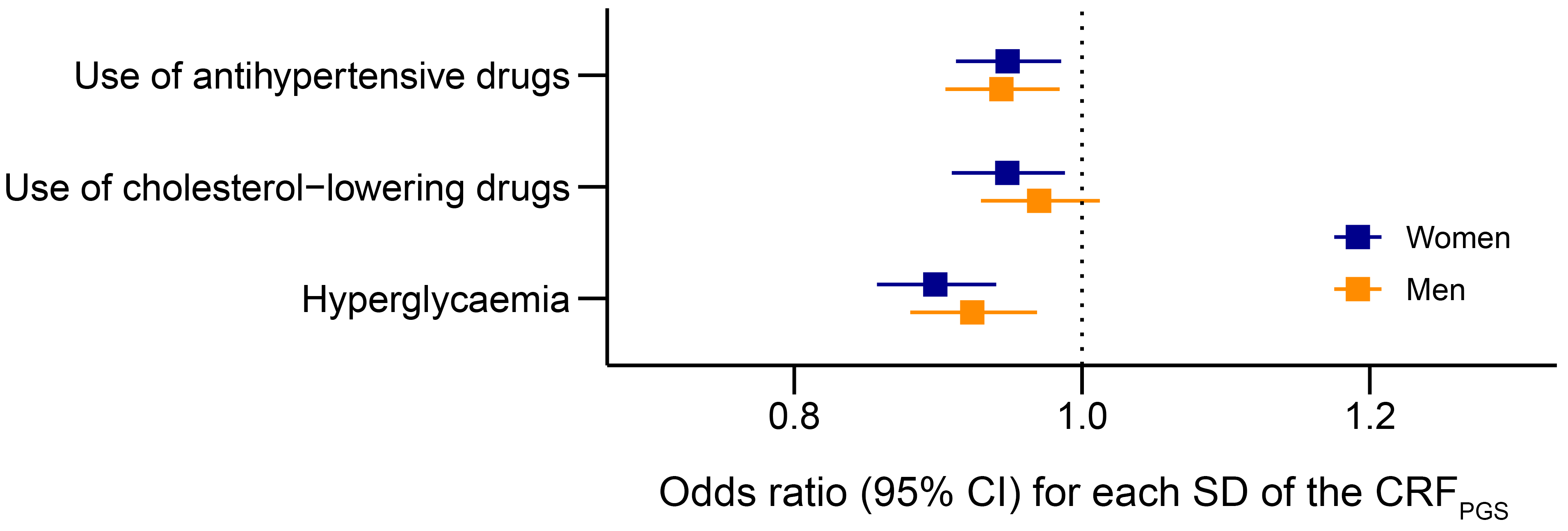

Supplement: Supplementary file 2 — Supplementary Material 2 [file 41598_2025_28894_MOESM2_ESM.png]

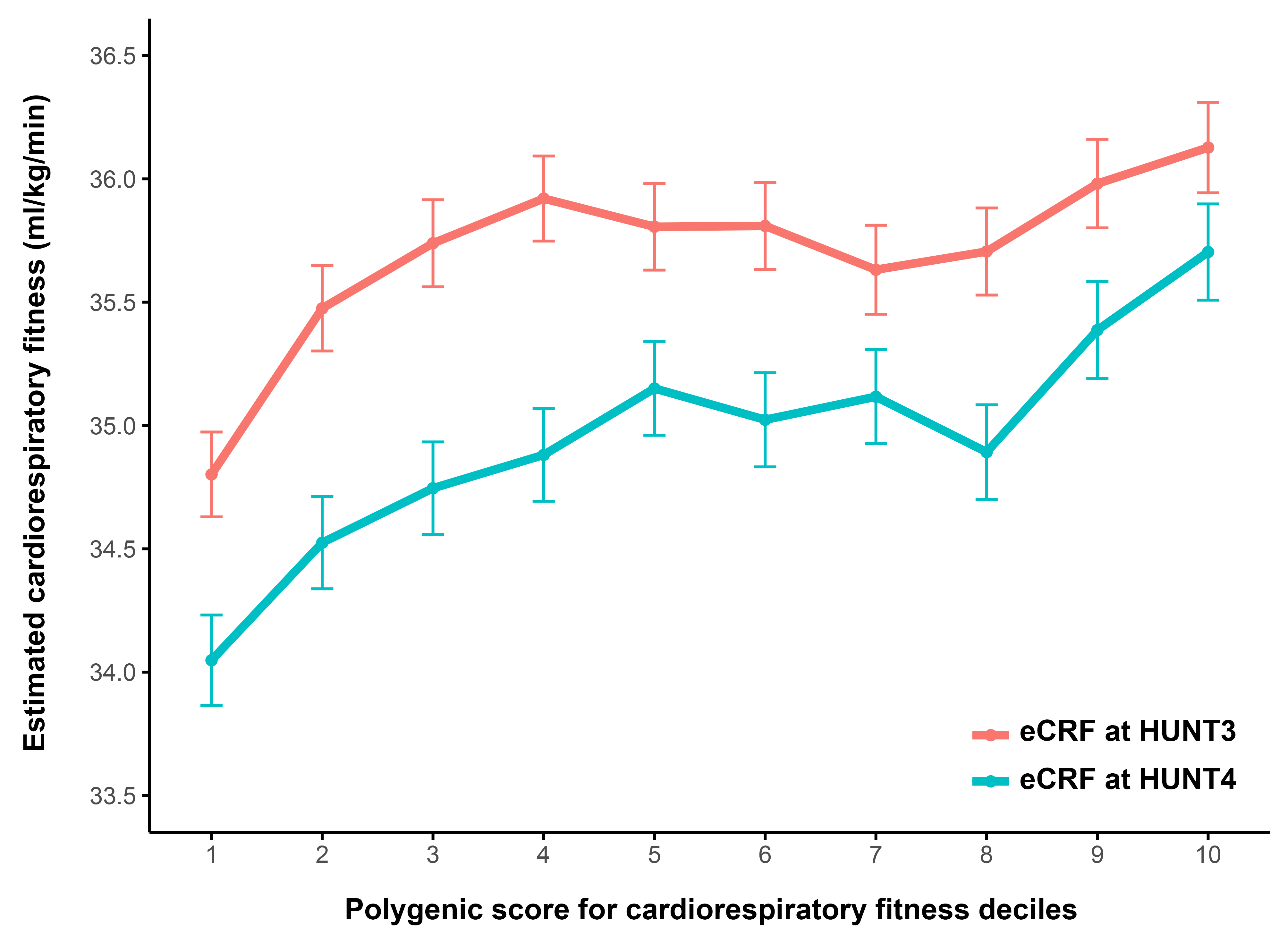

Supplement: Supplementary file 3 — Supplementary Material 3 [file 41598_2025_28894_MOESM3_ESM.png]

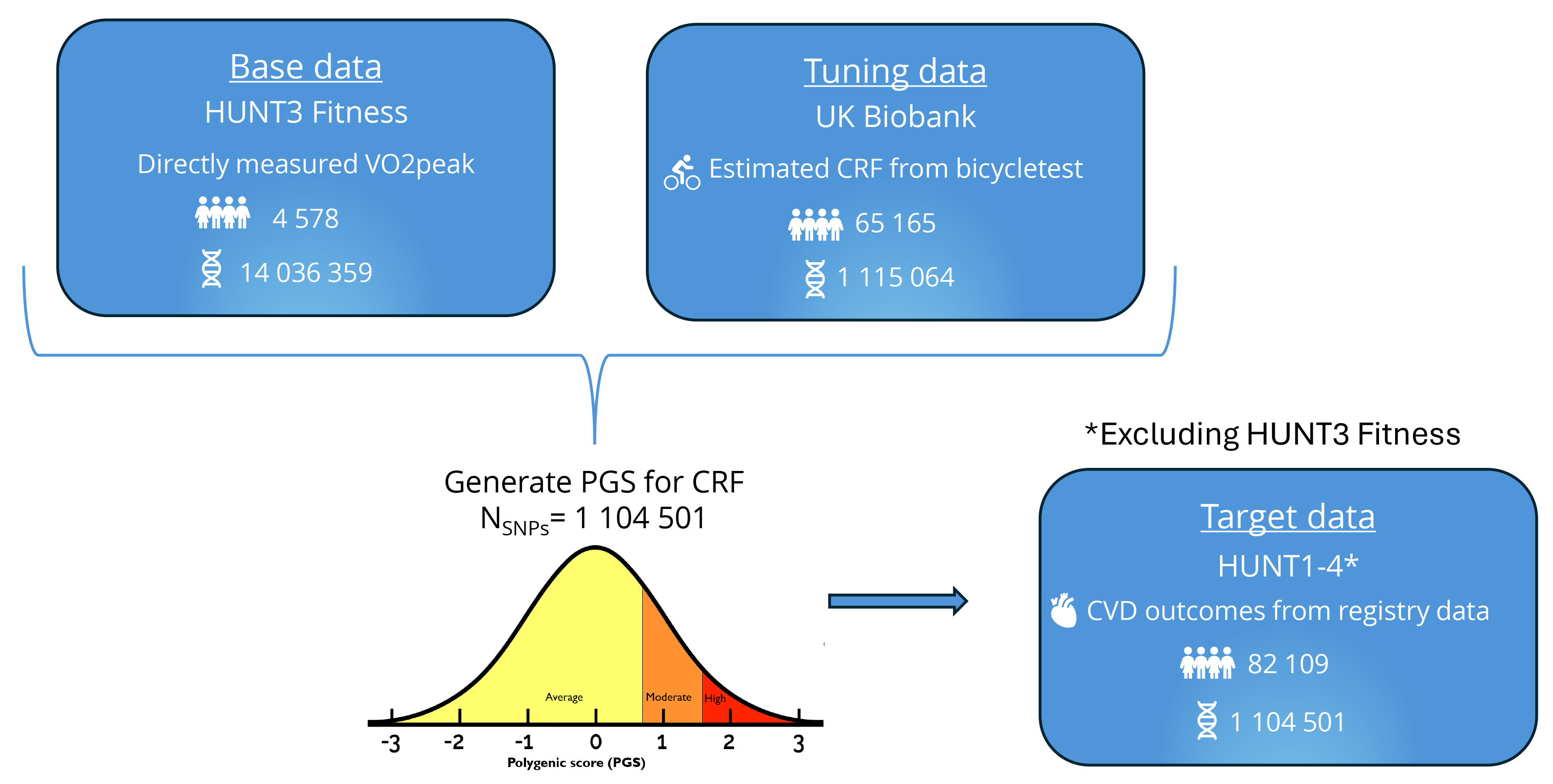

Supplement: Supplementary file 4 — Supplementary Material 4 [file 41598_2025_28894_MOESM4_ESM.png]
