# Supplementary material for: Polygenic prediction of cardiorespiratory fitness in the Trøndelag health study (HUNT)
Source: Sci Rep. 2025 Dec 16;16:184. doi: 10.1038/s41598-025-28894-7 (PMC12764957; doi:10.1038/s41598-025-28894-7)
Supplement: Supplementary file 5 — Supplementary Material 5 [file 41598_2025_28894_MOESM5_ESM.docx]

**Supplementary material**

**Polygenic Prediction of Cardiorespiratory Fitness in the Trøndelag Health Study (HUNT)**

Karsten Øvretveit^1,2,†,*^, Marie Klevjer^3,4,†,*^, Ben M. Brumpton^1,5^, Ulrik Wisløff^3^, Kristian Hveem^1,4^, Anja Bye^3,4^

^1^ HUNT Center for Molecular and Clinical Epidemiology (MCE), Department of Public Health and Nursing, Norwegian University of Science and Technology (NTNU), Trondheim, Norway.

^2^ Department of Education and Sports Science, University of Stavanger, Stavanger, Norway.

^3^ Cardiac Exercise Research Group (CERG), Department of Circulation and Medical Imaging,

Norwegian University of Science and Technology (NTNU), Trondheim, Norway.

^4^ Department of Cardiology, St. Olavs Hospital, Trondheim University Hospital, Trondheim, Norway

^5^ HUNT Research Centre, Department of Public Health and Nursing, Norwegian University of Science and Technology (NTNU), Levanger 7600, Norway

^†^ These authors contributed equally

**Table of contents**

Genotyping 2

Polygenic scores 2

Disease outcomes 3

Self-reported conditions 4

Physical activity index 4

References 5

# Genotyping

The Trøndelag Health Study (HUNT) cohorts were genotyped using one of four different Illumina HumanCoreExome arrays (HumanCoreExome12 v1.0, HumanCoreExome12 v1.1, UM HUNT Biobank v1.0 and UM HUNT Biobank v2.0)^1^. Samples from HUNT1-3 were imputed using Minimac3 (v2.0.1, http://genome.sph.umich.edu/wiki/Minimac3)^2^ with default settings (2.5 Mb reference-based chunking with 500kb windows) and the HUNT-WGS customized Haplotype Reference consortium release 1.1 (HRC v1.1) for autosomal variants and HRC v1.1 for chromosome X variants^3^. Samples from HUNT4 were phased with Eagle v2.4.1 (https://alkesgroup.broadinstitute.org/Eagle/) and imputed with the Positional Burrows-Wheeler Transform (PBWT) (https://github.com/richarddurbin/pbwt). More information about HUNT is available on the project’s website: https://www.ntnu.edu/hunt

# Polygenic scores

Multiple polygenic scores (PGS) for cardiorespiratory fitness (CRF) were developed using, PRS-CS, a Bayesian regression framework with continuous shrinkage priors^4^. We used summary statistics from the HUNT3 Fitness Study^5^ and an independent cohort from the UK Biobank (*n* = 65 165) to test genotype-phenotype associations before identifying the PGS with the highest effect size (**Table S1**).

**Table S1.** Polygenic score performance in the UK Biobank

| **Method** | **Tuning parameter** | **N SNPs in score** | **Correlation coefficient** |
| --- | --- | --- | --- |
| PRS-CS | Auto | 1 115 064 | 0.00374006 |
| PRS-CS | 1 | 1 115 064 | 0.01169925 |
| PRS-CS | 0.1 | 1 115 064 | 0.00978700 |
| PRS-CS | 0.01 | 1 115 064 | 0.00715169 |
| PRS-CS | 0.0001 | 1 115 064 | 0.00641753 |
| PRS-CS | 0.000001 | 1 115 064 | 0.00155524 |

# Disease outcomes

Disease outcomes were defined using the 10th revision of the International Statistical Classification of Diseases and Related Health Problems (ICD-10) (**Table S2**).

**Table S2.** Definition of outcomes using the 10th revision of the International Statistical Classification of Diseases and Related Health Problems (ICD-10)

| Outcome | ICD-10 code(s) |
| --- | --- |
| Cardiovascular disease | I11.0, I11.9, I13.0, I13.1, I13.2, I13.9, I20.0, I20.1, I20.8, I20.9, I21.0, I21.01, I21.02, I21.03, I21.0a, I21.0b, I21.1, I21.11, I21.13, I21.1a, I21.2, I21.21, I21.3, I21.31, I21.32, I21.3b, I21.4, I21.41, I21.42, I21.4a, I21.4b, I21.9, I21.91, I21.92, I21.9a, I24.8, I24.9, I25.0, I25.1, I25.2, I25.3, I25.4, I25.5, I25.6, I25.8, I25.9,I26.0, I26.9, I27.0, I27.2, I27.8, I27.9, I34.0, I34.1, I34.2, I34.8, I34.9, I35.0, I35.1, I35.2, I35.8, I35.9, I36.0, I36.1, I36.2, I36.8, I36.9, I37.0, I37.1, I37.8, I37.9, I42.0, I42.1, I42.2, I42.9, I44.0, I44.1, I44.2, I44.3, I44.4, I44.5, I44.6, I44.7, I45.0, I45.1, I45.2, I45.3, I45.4, I45.5, I45.6, I45.8, I45.9, I46.0, I46.1, I46.9, I47.0, I47.1, I47.2, I47.9, I48, I48.0, I48.1, I48.2, I48.3, I48.4, I48.9, I49.0, I49.1, I49.2, I49.3, I49.4, I49.5, I49.8, I49.9, I50.0, I50.1, I50.9, I51.0, I51.1, I51.2, I51.3, I51.4, I51.5, I51.6, I51.7, I51.8, I51.9, I60.0, I60.1, I60.2, I60.3, I60.4, I60.5, I60.6, I60.7, I60.8, I60.9, I61.0, I61.1, I61.2, I61.3, I61.4, I61.5, I61.6, I61.8, I61.9, I62.0, I62.1, I62.9, I63.0, I63.1, I63.2, I63.3, I63.4, I63.5, I63.6, I63.8 I63.9, I64, I65.0, I65.1, I65.2, I65.3, I65.8, I65.9, I66.0, I66.1, I66.2, I66.3, I66.4, I66.8, I66.9, I67.0, I67.1, I67.2, I67.3, I67.4, I67.5, I67.6, I67.7, I67.8, I67.9, I68.0, I70.0, I70.00, I70.01, I70.1, I70.10, I70.2, I70.20, I70.21, I70.8, I70.80, I70.81, I70.9, I70.90, I70.91, I71.0, I71.1, I71.2, I71.3, I71.4, I71.5, I71.6, I71.8, I71.9, I72.0, I72.1, I72.2, I72.3, I72.4, I72.5, I72.6, I72.8, I72.9, I73.9, I74.0, I74.1, I74.2, I74.3, I74.4, I74.5, I74.8, I74.9, I82.2, I82.3, I82.8, I82.9 |
| Myocardial infarction | I21.0, I21.01, I21.02, I21.03, I21.0a, I21.0b, I21.1, I21.11, I21.13, I21.1a, I21.2, I21.21, I21.3, I21.31, I21.32, I21.3b, I21.4, I21.41, I21.42, I21.4a, I21.4b, I21.9, I21.91, I21.92, I21.9a |
| Stroke | I60.0, I60.1, I60.2, I60.3, I60.4, I60.5, I60.6, I60.7, I60.8, I60.9, I61.0, I61.1, I61.2, I61.3, I61.4, I61.5, I61.6, I61.8, I61.9, I62.0, I62.1, I62.9, I63.0, I63.1, I63.2, I63.3, I63.4, I63.5, I63.6, I63.8, I63.9, I64 |
| Heart failure | I50.0, I50.1, I50.9 |
| Hypertrophic cardiomyopathy | I42.1, I42.2 |
| Atrial fibrillation | I48 |
| Hypertension | I10 |

# Self-reported conditions

Hyperglycemia and medication use was assessed using questionnaire data (**Table S3**). The medication questions were part of clusters with the first question asking if they are currently using prescription drugs.

**Table S3.** Self-reported disease-related outcomes

| **Outcome** | **Question** |
| --- | --- |
| Verified hyperglycemia | *“Has it ever been verified that you had high blood sugar (hyperglycaemia)?”* |
| On lipid-lowering medication | *“If yes, do you currently use prescription medication?”* |
| On antihypertensives | *If yes, do you currently use prescription medication?* |

# Physical activity index

The physical activity (PA) index is calculated based on self-reported questionnaire data. Three questions regarding PA were used to calculate the PA index. The number in the brackets behind every response option was multiplied for each question and constitutes the PA index. Question 1: “How frequently do you exercise?” with the response options: ”Never” (0), “Less than once a week” (0), “Once a week” (1), “2–3 times per week” (2.5), and “Almost every day” (5). Question 2: “If you exercise as frequently as once or more times a week: How hard do you push yourself?” with response options: “I take it easy without breaking a sweat or losing my breath” (1), “I push myself so hard that I lose my breath and break into sweat” (2), and “I push myself to near exhaustion” (3). Question 3: “How long do each session last?” with the response options: “Less than 15 minutes” (0.1), “16–30 minutes” (0.38), “30 minutes to 1 hour” (0.75) and “More than 1 hour” (1.0).

# References

1 Brumpton, B. M. *et al.* The HUNT study: A population-based cohort for genetic research. *Cell Genomics* **2**, doi:10.1016/j.xgen.2022.100193 (2022).

2 Das, S. *et al.* Next-generation genotype imputation service and methods. *Nat. Genet.* **48**, 1284-1287, doi:10.1038/ng.3656 (2016).

3 McCarthy, S. *et al.* A reference panel of 64,976 haplotypes for genotype imputation. *Nat. Genet.* **48**, 1279-1283, doi:10.1038/ng.3643 (2016).

4 Ge, T., Chen, C.-Y., Ni, Y., Feng, Y.-C. A. & Smoller, J. W. Polygenic prediction via Bayesian regression and continuous shrinkage priors. *Nat. Commun.* **10**, 1776, doi:10.1038/s41467-019-09718-5 (2019).

5 Klevjer, M. *et al.* Genome-Wide Association Study Identifies New Genetic Determinants of Cardiorespiratory Fitness: The Trøndelag Health Study. *Med. Sci. Sports Exerc.* **54**, 1534-1545, doi:10.1249/mss.0000000000002951 (2022).
